# Silver Nanoparticles Synthesized by Using *Bacillus cereus* SZT1 Ameliorated the Damage of Bacterial Leaf Blight Pathogen in Rice

**DOI:** 10.3390/pathogens9030160

**Published:** 2020-02-26

**Authors:** Temoor Ahmed, Muhammad Shahid, Muhammad Noman, Muhammad Bilal Khan Niazi, Faisal Mahmood, Irfan Manzoor, Yang Zhang, Bin Li, Yong Yang, Chengqi Yan, Jianping Chen

**Affiliations:** 1 State Key Laboratory of Rice Biology, Institute of Biotechnology, Zhejiang University, Hangzhou 310058, China; temoorahmed248@gmail.com (T.A.); nomansiddique834@gmail.com (M.N.); 0618151@zju.edu.cn (Y.Z.); libin0571@zju.edu.cn (B.L.); 2 Department of Bioinformatics and Biotechnology, Government College University, Faisalabad 38000, Pakistan; rana.arfan84@gmail.com; 3 School of Chemical and Materials Engineering (SCME), National University of Sciences & Technology (NUST), Sector H-12, Islamabad 44000, Pakistan; m.b.k.niazi@scme.nust.edu.pk; 4 Department of Environmental Science and Engineering, Government College University, Faisalabad 38000, Pakistan; fslagronomy@hotmail.com; 5 State Key Laboratory for Quality and Safety of Agro-Products, Institute of Virology and Biotechnology, Zhejiang Academy of Agricultural Sciences, Hangzhou 310021, China; yangyong@zaas.ac.cn; 6 Institute of Plant Virology, Ningbo University, Ningbo 315211, China; yanchengqi@163.com

**Keywords:** antimicrobial activity, BLB, nanotechnology, rice, silver nanoparticles, *B. cereus*

## Abstract

Amongst serious biotic factors deteriorating crop yield, the most destructive pathogen of rice is *Xanthomonas oryzae* pv. *oryzae* (*Xoo*), which causes bacterial leaf blight (BLB) disease. This study involved targeted use of biogenic silver nanoparticles (AgNPs) to control BLB in order to cope with the disadvantages of chemical disease control. AgNPs were biologically synthesized from natively isolated *Bacillus* cereus strain SZT1, which was identified through 16S rRNA gene sequence analysis. Synthesis of AgNPs in bacterial culture supernatant was confirmed through UV-VIS spectroscopy. Fourier transform infrared spectroscopy (FTIR) confirmed that the existence of AgNPs was stabilized with proteins and alcoholic groups. X-ray diffraction (XRD) data revealed the crystalline nature and imaging with scanning electron microscopy (SEM) and transmission electron microscopy (TEM), showing the spherical shape of AgNPs with particle sizes ranging from 18 to 39 nm. The silver presence in AgNPs was further confirmed by energy dispersive spectra. Biogenic AgNPs showed substantial antibacterial activity (24.21 ± 1.01 mm) for *Xoo*. In a pot experiment, AgNPs were found to be effective weapons for BLB by significantly increasing the plant biomass with a decreased cellular concentration of reactive oxygen species and increased concentration of antioxidant enzyme activity.

## 1. Introduction

Rice (*Oryza sativa* L.) is one of the world’s most important staple foods and is cultivated on a large acreage of arable land [1]. Approximately, 90% of the world’s rice is grown in Asia, and China remains among the world’s largest rice-producing countries [2,3]. By 2050, the human population is anticipated to increase from approximately 7.5 to 9.5 billion worldwide [4]. Thus, to meet future rice trade and demands, rice producers must increase the rice yield to feed the growing population. On the other hand, agricultural systems are being continuously affected by various types of biotic and abiotic stresses. Plant diseases of agricultural farms are the major biotic constraints affecting crop productivity, and thus, may lead to global food crisis [5]. The most important bacterial pathogen of rice is *Xanthomonas oryzae* pv. *oryzae* (*Xoo*), which causes bacterial leaf blight (BLB) disease [6,7].

As a result of the climate change and increase in temperature, infestation of the BLB disease caused by the *Xoo* pathogen is increased, particularly in China and Pakistan due to the favorable environmental conditions, resulting in great damage to rice crop [8]. Chemical control in the form of numerous chemicals and antibiotics has been used for treatment of BLB. Chemical disease control is expensive and harmful for the climate, humans, and agricultural produce, and also leads to pathogen resistance [9]. Cultivation of resistant rice cultivars developed through breeding programs is another effective approach to manage the BLB disease. Unfortunately, this technique is frequently ineffective due to the rapid adaptations of the pathogenic populations [5]. In the past few years, a broad spectrum of experiments have been carried out on the use of bacterial antagonists, particularly *Lysobacter antibioticus* [10], *Pseudomonas* spp. [11], and *Bacillus* spp. [12] for the biological control of BLB disease.

Recently, nanotechnology has emerged as a promising area of research and has numerous applications in agriculture, especially the use of nanoparticles (NPs) to control plant diseases and improve agricultural yield [13,14]. Nanoparticles are efficient, low-cost, and environmentally friendly alternatives to materials, as they process with superior efficiency, a high reaction rate, and a large surface/mass ratio. There are currently more than 1800 consumer products containing NPs [15]. A large volume of research has been developed over the past decades in order to create more efficient, resistant, and better performing nano-materials than the macro-material products currently available in the market [16]. 

Microbe-based biosynthesis of NPs is beneficial as compared to chemical and physical methods due to their non-toxic, eco-friendly, cost-efficient, and more stable nature [17,18,19]. Biogenic silver nanoparticles (AgNPs) have been given significant attention due to their unique biological, physico-chemical, and antimicrobial activity properties in controlling plant pathogens [20,21]. Various studies have been reported that AgNPs are able to suppress many bacterial phytopathogens, such as *Acidovorax oryzae* strain RS-2 [22], *Clavibacter michiganensis* subspecies *michiganensis* [23], and *Pseudomonas syringae* pv. *tomato* DC 3000 [24]. Green AgNPs, synthesized by using bacteria of the genus *Bacillus*, including *Bacillus megaterium*, *Bacillus cereus*, *Bacillus amyloliquefaciens*, *Bacillus flexus*, and *Bacillus subtilis*, have been widely studied and have a sustainable nature [18,25]; however, none of these *Bacillus* spp. have reported characteristic silver-resistance for AgNPs biosynthesis, the mechanism of resistance to Ag and subsequence antimicrobial efficacy. Moreover, wastewater-contaminated sites, due to presence of huge concentrations of metals may serve as niches of bacteria with inherent metal NP biosynthesis potential.

In order to evaluate the above-mentioned components, an AgNO_3_-resistant strain *Bacillus cereus* SZT1 was isolated and used for biological synthesis of the AgNPs. Furthermore, the study characterized the biologically synthesized AgNPs with strong in vitro and in vivo antibacterial activity against *Xoo*. Overall, the results from this study provided cost-efficient and environmentally friendly alternatives for control of BLB disease in rice.

## 2. Results 

### 2.1. Soil Analysis

**Figure 1:**
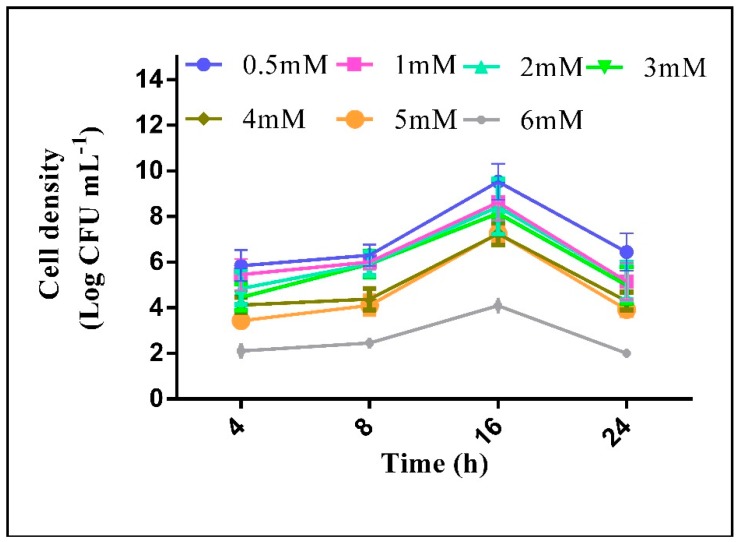
Growth curves of *B. cereus* SZT1 in the presence of different AgNO_3_ concentrations.

Heavy metal contamination and other physicochemical properties of wastewater-contaminated soil were measured to ensure the inherent capacity of isolated strain for metal tolerance under natural conditions (Table 1). The soil was sandy clay in texture with high electrical conductivity (EC) and pH. The nutritional profile indicated that soil contained N, available P, and K in low amounts. The soil was found to exhibit high metal content of Ag, Zn, Cd, Ni, and Pb, whereas it was found to be poor in organic content (Table 1). The purified strain SZT1, isolated in this study, was found to tolerate AgNO_3_ up to 5 mM under in vitro conditions. The strain showed optimal growth response (approximately 7.5 to 9.5 log CFU mL^−1^) to various AgNO_3_ concentrations (Figure 1), however, the growth in the culture medium dropped significantly at 6 mM of AgNO_3_, and no growth was measured at 7 mM and 8 mM of AgNO_3_ concentration.

### 2.2. Identification and Phylogenetic Analysis of B. cereus SZT1

**Figure 2:**
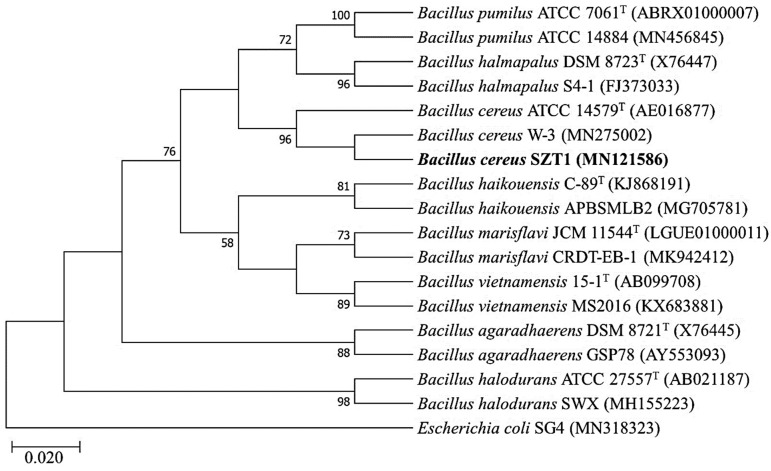
Phylogenetic tree of *B. cereus* SZT1 with the type strains of genus *Bacillus*. The evolutionary history was inferred using the maximum likelihood (ML) method. The percentages (≥50%) of replicate trees in which the associated taxa clustered together in the bootstrap test (1000 replicates) are shown. Evolutionary distances were computed using the Timura–Nei model and are in units of the number of base substitutions per site.

The 16S rRNA gene of strain SZT1 was successfully sequenced and submitted to the National Center for Biotechnology Information (NCBI) database (accession no. MN121586). BLASTn analysis of the 16S rRNA gene sequence revealed that strain SZT1 had more than 99.53% sequence similarity with *B. cereus* BM2 (KY773607) and *B. cereus* BD7 (KY773599), whereas 99.53% similarity was found with *B. cereus* ATCC 14579^T^ (AE016877) when searched using EzBioCloud. Similarly, in the SILVA database, the sequence also showed similarity with *B. cereus* (LZOD01000018). Furthermore, strain SZT1 formed one cluster with *B. cereus* ATCC 14579^T^ (AE016877) and was well separated from the other species of the *Bacillus* genus in the phylogenetic tree (Figure 2). Therefore, it was able to be inferred that strain SZT1 should be designated as *B. cereus*.

### 2.3. Biosynthesis of AgNPs by B. cereus SZT1

The bacterial strain SZT1 was able to synthesize AgNPs at 5 mM concentration of AgNO_3_. At concentrations lower and greater than 5 mM, the color change (bioreduction process) was found to be less than that of 5 mM concentration. The strain SZT1 showed a reddish-brown color of its supernatant after the addition of 5 mM AgNO_3._

### 2.4. Antibacterial Activity of Biogenic AgNPs

**Figure 3:**
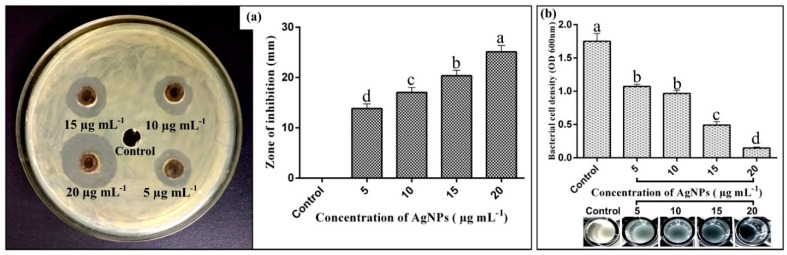
Antibacterial activity of silver nanoparticles (AgNPs) synthesized from *B. cereus* SZT1 in various concentrations against *Xanthomonas oryzae* pv. *oryzae* (*Xoo*). (**a**) measurement of antibacterial activity through well-diffusion assay; (**b**) measurement of antibacterial activity through inhibition of bacterial cell density.

Results of antibacterial activity indicated that AgNP suspension at four different concentrations (5, 10, 15, and 20 µg mL^−1^) significantly suppressed growth of rice pathogen *Xoo* as compared with non-treated control (Figure 3a). The inhibitory effect on bacterial growth increased with the increase of the concentration of AgNPs. The largest antibacterial activity was observed in AgNP suspension at 20 µg mL^−1^, which exhibited the maximum diameter of the inhibition zone (25.11 ± 0.35 mm). In vitro minimum inhibitory concentration (MIC) results revealed that AgNPs significantly inhibited the growth of *Xoo* after 48 h of incubation. Indeed, the inhibitory effect on bacterial growth was increased with the increase of the concentration of AgNPs. The AgNPs suspension at four different concentrations (5, 10, 15, and 20 µg mL^−1^) caused (38.85%, 45.14%, 57.71%, 91.42%) reduction of *Xoo* at OD600, respectively (Figure 3b). Hence, the result revealed that biologically synthesized AgNPs have great potential in controlling bacterial leaf blight of rice.

### 2.5. Characterization of Biogenic AgNPs

**Figure 4:**
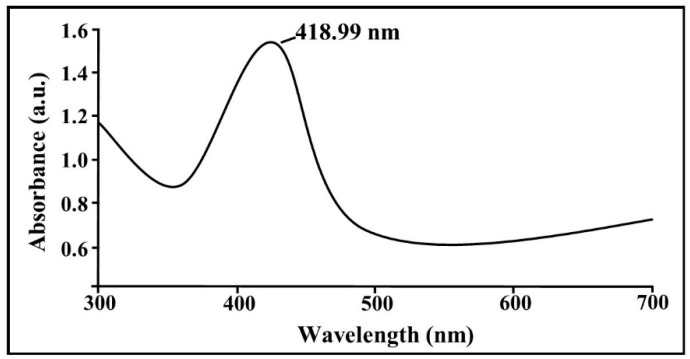
UV-VIS spectrum of cell free supernatant containing biogenic AgNPs synthesized by using *B. cereus* SZT1 after 24 h. The absorption spectrum of biogenic AgNPs showed a strong peak at 418.99 nm.

**Figure 5:**
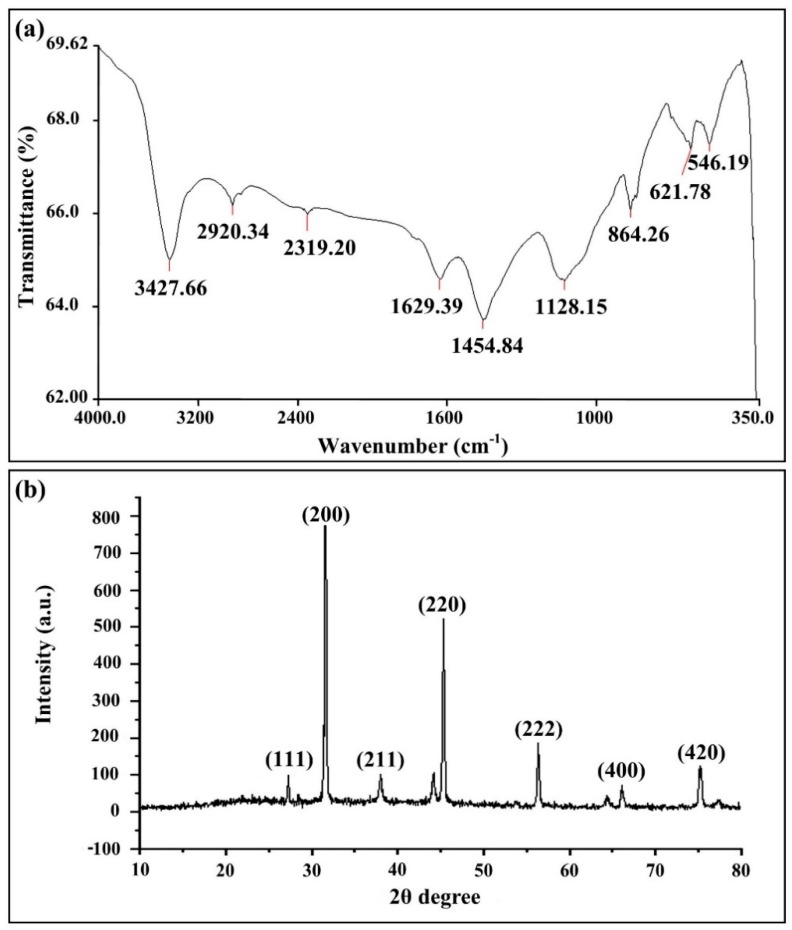
Characterization of the biogenic AgNPs synthesized from *B. cereus* SZT1. (**a**) FTIR spectra of the biogenic AgNPs synthesized from *B. cereus* SZT1 in the wavelength range of 350-4000 cm^−1^; (**b**) XRD spectrum of biogenic AgNPs synthesized from *B. cereus* SZT1.

The presence of the biogenic AgNPs in the supernatant of *B. cereus* SZT1 was confirmed by a peak in the UV region at 418.99 nm on the basis of the UV/VIS spectral analysis (Figure 4). The infrared spectra of biogenic AgNPs produced from *B. cereus* SZT1 showed peaks at 3427.66, 2920.34, 2319.20, 1629.39, 1454.84, and 1128.15 cm^−1^. The strong peak at 3427.66 cm^−1^ and medium peak at 2920.34 cm^−1^ were due to the broad absorption of the hydroxyl (OH^−^) group of alcohol and a C-H stretching group of alkane, respectively. The peaks at 2319.20 and 1629.39 cm^−1^ were due to stretching of O=C=O and a C=C stretching group of alkene, respectively, whereas, the strong peaks at 1454.84 cm^−1^ and 1128.15 cm^−1^ were due to the N-O stretching group of a nitro compound and stretching of a C-N group of amine, respectively (Figure 5a).

The X-ray diffraction data of biogenic AgNPs of *B. cereus* SZT1 showed peak positions of 2θ degree values ranging from 20^θ^ to 90^θ^ at 27.19°, 31.52°, 37.93°, 45.26°, 56.30°, and 76.34°, which corresponded to 111, 200, 220, 222, 400, and 420 respective planes of cubic silver. Thus, data were in accordance with the standard diffraction values of AgNPs (JCPDS card #: 04-0783). Biogenic AgNPs were found to have an average particle size of 22.96 nm, according to Debye–Sherrer’s equation (Figure 5b).

**Figure 6:**
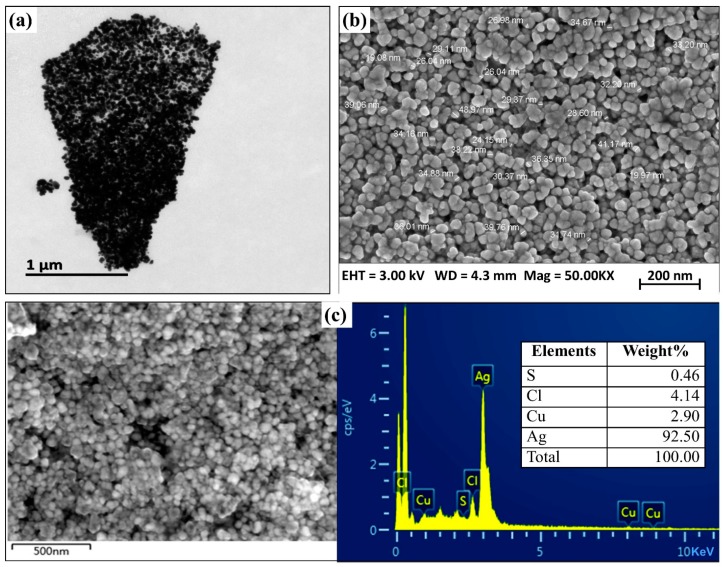
Characterization of the biogenic AgNPs synthesized from *B. cereus* SZT1. (**a**) Transmission electron microscopy; (**b**) scaning electron microscopy; (**c**) EDS spectrum.

SEM and TEM images provided surface morphology details of biogenic AgNPs. SEM images at 50,000× magnitude scale were collected and studied. The results of the SZT1-AgNPs sample showed spherical shapes and particle sizes ranging from 18 nm to 39 nm (Figure 6a,b). Presence of elemental silver was further confirmed by energy-dispersive spectrum (EDS) analysis. The EDS spectrum showed the peaks of biogenic AgNP elements, consisting of silver (92.50%), chlorine (4.14%), copper (2.90%), and sulphur (0.46%) (Figure 6c).

### 2.6. Effect of AgNPs on Lesion Length and Inhibition Rate of Xoo

Effects of AgNP suspension at four different concentrations (25, 50, 75, and 100 mg L^−1^) on the lesion length of BLB showed significant reduction as compared with non-treated control plants (Table 2). The lesion length was decreased with the increase of the concentration of AgNPs. The maximum inhibition rate was observed in rice plants treated with AgNP suspension at 100 mg L^−1^, (72.51%). Hence, the result revealed that the biogenic AgNPs have great potential in bio-controlling BLB in rice fields.

### 2.7. Effect of AgNPs on the Growth of Healthy and Diseased Rice Plants

In the absence of the bacterial pathogen, no significant difference was found in the growth parameters, except that root length that was found to be increased by AgNPs at 75 and 100 mg L^−1^ and that root dry weight was increased by AgNPs at 75 mg L^−1^. Indeed, AgNPs at 75 and 100 mg L^−1^ caused a 12.87% and 16.18% increase in root length of healthy rice plants, respectively, whereas the spray of AgNPs at 100 mg L^−1^ resulted in a 11.23% increase in root dry weight compared to non-treated healthy plants (Table 3).

Compared to the healthy plants, inoculation of the bacterial pathogen caused a significant reduction in the growth parameters of rice plants. The infected plants showed a reduction in root and shoot length (36.21% and 19.82%), and root and shoot fresh (34.14% and 32.10%) and dry weight (48.75% and 28.62%), respectively, in comparison with healthy control plants. In contrast, AgNPs of 25, 50, 75, and 100 mg L^−1^ caused a 21.61%, 38.33%, 55.91%, and 89.91% increase in root length, an 8.69%, 20.93%, 23.24%, and 25.55% increase in shoot length, and a 9.43%, 16.04%, 34.54%, and 46.48% increase in shoot fresh weight, respectively, compared to the pathogen control (Table 3). Furthermore, root fresh weight, root dry weight, and shoot dry weight were unaffected by AgNPs of 25 mg L^−1^. However, AgNPs of 50, 75, and 100 mg L^−1^ caused 16.14%, 38.76%, and 49.64% increases in root fresh weight, 40.20%, 57.92%, and 90.11% increases in root dry weight, and 11.05%, 24.28%, and 26.71% increases in shoot dry weight, respectively, compared to the pathogen control (Table 3).

### 2.8. Effect of AgNPs on the Physiology of Healthy and Diseased Rice Plants

The physiological parameter results showed that the inoculation of bacterial pathogen significantly increased the malondialdehyde (MDA) and H_2_O_2_ contents of healthy rice plants. However, the AgNPs at four different concentrations (with maximum effect at 100 mg L^−1^ AgNP level) were able to decrease the oxidative damage to the healthy and diseased rice plants due to reactive oxygen species (ROS) such as the MDA and H_2_O_2_ contents, except that the MDA content was unaffected by the AgNPs of 25 mg L^−1^ in diseased plants. Indeed, in the absence of a bacterial pathogen, the AgNPs significantly decreased the MDA content by 62.34% and H_2_O_2_ content by 33.31% up to 100 mg L^−1^ concentration as compared to the healthy control. In the presence of bacterial pathogen, the AgNPs at 100 mg L^−1^ caused a 48.63% and 60.12% decrease in MDA and H_2_O_2_ content, respectively, in contrast with infected control plants (Table 4).

In the absence of a bacterial pathogen, the level of antioxidants was significantly improved by AgNPs, with a maximum boost in the antioxidants at 100 mg L^−1^ NP level. Proline and catalase (CAT) content was unaffected by the AgNPs at 25 mg L^−1^, however, the AgNPs at 100 mg L^−1^ caused 59.24%, 59.76%, 45.61%, and 141.96% increases in the proline content, CAT, peroxidase (POD), and total phenolics of heathy rice plants, respectively, as compared to their control treatment. However, the difference in the means of proline and CAT content was found to be non-significant as compared to the healthy control at 25 mg L^−1^ AgNPs. Similarly, no significant difference in the proline content and total phenolics between the healthy and diseased rice plants was found, whereas the inoculation of bacterial pathogen significantly reduced CAT and POD content of healthy rice plants. In the presence of a bacterial pathogen, the proline content was significantly increased by the AgNPs at 100 mg L^−1^, but not affected by AgNPs at the other three concentrations, whereas the CAT content was unaffected by the AgNPs at 25 mg L^−1^, but was significantly increased by the AgNPs at the other three concentrations. Indeed, the AgNPs at 100 mg L^−1^ caused a 23.14% and 59.97% increase in the proline and CAT content, respectively, compared to the pathogen control; similarly, AgNPs significantly increased the POD and total phenolics up to a maximum extent by 53.14% and 113.51% at 100 mg L^−1^ AgNPs, respectively, as compared to pathogen control (Table 4). 

In general, the result of this study revealed that the physiological parameters of rice plants were differentially affected by the inoculation of bacterial pathogen; indeed, the contents of MDA and H_2_O_2_ were increased, the contents of CAT and POD were reduced, and the levels of proline and total phenolics were unaffected by the pathogen infection. Similarly, the differential effects of AgNPs were observed on the diseased rice plants. Compared to the pathogen control, the content of MDA was unaffected by AgNPs at 25 mg L^−1^, but was significantly reduced by AgNPs at the other three concentrations. AgNPs significantly reduced the content of H_2_O_2_ at all concentrations, whereas the proline content was increased by AgNPs at 100 mg L^−1^, but unaffected by the other three concentrations. The CAT content and the total phenolics level were unaffected by AgNPs at 25 mg L^−1^, but significantly increased by the other three concentrations. AgNPs significantly increased the POD content at all concentrations (Table 4).

## 3. Discussion 

In this study, the AgNPs biosynthesized by using bacterial strain *B. cereus* SZT1 were biologically synthesized and subsequently evaluated as antibacterial agents against rice bacterial pathogen. The silver-resistant bacterial strain SZT1 was isolated from wastewater-contaminated soil and evaluated for the biosynthesis of AgNPs under different concentration of AgNO_3_. The reason for the selection of the wastewater-contaminated site for bacterial isolation was its high probability of the presence of metal-tolerant bacteria capable of AgNPs synthesis. SZT1 was found to be resistant to more than 5 mM of AgNO_3_. Various bacterial strains have been shown to have resistance against silver ions, and this resistance is attributed genetically to their plasmids and genes [26]. For instance, *Klebsiella pneumonia* and *Enterobacter* cloacae were found to be resistant to up to 5500 µM silver [27]. Similarly, Andriano et al. [28] reported the AgNPs synthesis potential and resistance of *Bacillus* spp. isolated from soil for AgNO_3_ up to 10 mM. The sequence analysis of the 16S rRNA gene of strain SZT1 revealed its identity as *B. cereus* [29,30]. The AgNP-containing supernatant of strain SZT1 showed a peak at 418.99 nm in UV/VIS spectral analysis. These results corroborate with Saravanan et al. [25] who confirmed the formation of AgNPs by the presence of the peak at 420 nm. Although some other *Bacillus* spp. have been reported for AgNPs biosynthesis, this study reports the significance of silver-resistant *Bacillus* strain SZT1 for synthesis and antibacterial potential against *Xoo*. Hence, investigation of genetic mechanisms of resistance to Ag and their correlation with green AgNP synthesis may be a future approach for devising sustainable plant disease control strategies.

Omole et al. [31] previously provided evidence that capping protein coat is essential for the stabilization of AgNPs synthesized from *B. subtilis*. Interestingly, the result of FTIR analysis in the current study revealed the presence of coating proteins, which have been regarded as being essential for the long-term stabilization of biogenic nano-materials. In agreement with the result of Kalishwaralal et al. [32], the crystalline nature of biogenic AgNPs as cubic silver lattice was confirmed by the different angles in 111, 200, 220, 222, 400, and 420 planes. In general, spherical morphology of the biosynthesized AgNPs are in line with those reported previously on the basis of the SEM and TEM images [31]. However, there was a slight difference in particle size and chemical composition of AgNPs between this study and other studies. For example, Ibrahim et al. [33] revealed that chemical composition of the size of biogenic AgNPs ranged from 25 to 50 nm and contained 91.80% silver. 

Results of in vitro antibacterial experiment showed that growth of rice pathogenic bacteria *Xoo* was significantly inhibited by AgNPs at four different concentrations; however, the inhibitory effect increased with the increase of the concentration of the AgNPs. Our results are comparable with Fouad et al. [34], who found a similar antibacterial activity of biogenic AgNPs against rice pathogen *Xoo*, whereas the highest zone of inhibition diameter of 23.33 mm was observed at 20 µg mL^−1^ concentration. In another study, Ibrahim et al. [33] reported that AgNPs synthesized from endophytic bacterium *Bacillus siamensis* strain C1 revealed a strong antibacterial effect against the rice pathogen *Xoo* with a 17.11 mm zone of inhibition. Similarly, the MIC results correlate with the findings of Masum et al. [22], who observed the maximum reduction of 67.43% at 30 µg mL^−1^ AgNP concentration against *Acidovorax oryzae* strain RS-2. Our previous studies indicated that inhibitory mechanism of biogenic AgNPs on rice bacterial pathogen might have been mainly due to the damage to the bacterial membrane, resulting in the release of genetic material and other cellular components of pathogen [34].

In agreement with the result of in vitro antibacterial activity, the biogenic AgNPs significantly reduced lesion length and damage of the bacterial leaf blight to rice plants in a pot experiment as compared to the control. In this study, the maximum reduction in lesion length and inhibition rate percentage was observed in rice plants treated with AgNP suspension at 100 mg L^−1^. Similarly, Degrassi et al. [35] observed that Fe_3_O_4_ and TiO_2_ NPs were able to significantly restrict the development of BLB lesion length as compared to the non-treated control in a pot experiment. Disease control potential of biogenic AgNPs, in the current study, at 100 mg L^−1^ (72.51% disease inhibition) was comparable to Nasir et al. [36] who reported 63%-92% BLB disease inhibition by the application of various antibiotics and fungicides. In another study, Chikte et al. [37] concluded that foliar application copper nanoparticles resulted in 90% and 15% reduction of bacterial blight of pomegranate at early and mature disease stage, respectively, under controlled conditions, whereas 20% disease inhibition was measured under field conditions. 

The growth-promoting effect of biogenic AgNPs was generally observed in diseased plants sprayed with different concentrations of nanoparticles, except root fresh weight (FW), root dry weight (DW), and shoot DW were unaffected by 25 mg L^−1^ AgNPs. In addition, the result of this study indicated that the growth-promoting effect depends on the concentration of biogenic AgNPs. These results clearly revealed the disease-suppressive effects of biogenic AgNPs, which were able to help pathogen-inoculated rice plants to thrive under stressful conditions of BLB disease. In agreement with these results, Salama [38] found increased growth of root and shoot in maize plants by biogenic AgNP application. In another study, Ejaz et al. [39] reported that AgNPs synthesized from plant source increased rice leaf area, leaf number, and leaf fresh and dry weight at a concentration of 75 mg L^−1^.

The physiological data revealed a similar trend of biogenic AgNPs in the plants, regardless of the presence or absence of the bacterial pathogen. In general, the biogenic AgNPs showed a protective effect on the healthy and diseased rice plants by increased antioxidant enzyme levels to modulate the negative effects of ROS. However, the protective content depended on the plant parameters and concentration of AgNPs. In general, the protective effect increased with the increase of AgNP concentration. In agreement with the result of this study, the authors of [40] reported that AgNPs were able to promote the nutrient uptake and cellular antioxidative system in rice plants under biotic and abiotic stress. Therefore, it can be inferred that the protective effect of AgNPs on rice plants from *Xoo* infection under controlled conditions might be mainly due to the activation of the antioxidative system, which relieved disease-affected plants from oxidative damage and thus improved the plant health. Although, it is well established that AgNPs demonstrate antibacterial effect by blocking the DNA replication and releasing the silver ions inside bacterial cells [41], further investigations are needed to establish the exact mode of action of biogenic AgNPs for BLB disease control.

## 4. Materials and Methods 

### 4.1. Origin of Strain B. cereus SZT1

The strain SZT1 was isolated from leather industry wastewater-contaminated soil adjacent to Kasur, Pakistan (31°19′55 N and 74°23′2 E) through dilution plate method as described by Somasegaran and Hoben [42], in the presence of 1 mM AgNO_3_ in the culture medium. The actual resistance of the bacterial strain SZT1 to different concentrations (0.5, 01, 02, 03, 04, 05, 06, 07, and 08 mM) of AgNO_3_ was confirmed by the determination of minimum inhibitory concentration (MIC) and growth curves by adding the same concentrations of AgNO_3_ in nutrient broth medium, constructed by measuring the cell density at different time intervals (4, 8, 16, 24 h) to determine the growth response of strain SZT1 to silver salt. The soil analysis for physicochemical parameters and measurement of heavy metal concentrations were necessary in order to access the extreme natural habitat of the potent strain SZT1. The soil samples of the sampling site were analyzed for various soil parameters by the standard procedures described in Ryan et al. [43]. The soil heavy metal analysis was carried out by atomic absorption spectrophotometer (Hitachi, Model 7JO-8024, Tokyo, Japan).

### 4.2. Identification of B. cereus SZT1

Genomic DNA of the bacterial strain SZT1 was isolated by the CTAB method [44] and measured by the Nano DropDrop 2000/2000c. This DNA was used as a template to amplify the 16S rRNA gene by means of primers fD1 5′-AGAGTTTGATCCTGGCTCAG-3′ and rD1 5′-AAGGAGGTGATCCAGCC-3′ [45]. The 16S rRNA gene was sequenced through Macrogen (South Korea) by Sanger’s method. The sequences were compared to others in a database by using the online BLASTn tool EzBioCloud and ACT tool of the SILVA database. The phylogenetic-analysis of the 16S rRNA sequence was carried out by using the MEGA 7.0 software package, and the phylogenetic tree was constructed by the maximum likelihood (ML) technique.

### 4.3. Extracellular Biosynthesis AgNPs

Pure culture of silver-resistant bacterial strain SZT1 was used to synthesize the AgNPs by extracellular supernatant-based biosynthesis mechanism according to the method of Fouad et al. [34]. The strain was aerobically grown in nutrient broth media amended with 1 mM AgNO_3_ at 28 ± 2 °C on an orbital-shaker at 150 rpm for 24 h. Cultures were centrifuged at 10,000× *g* for 5 min to collect the supernatant. The AgNP synthesis was investigated at seven different concentrations (0.5, 1, 3, 5, 7, 10, and 15 mM) of AgNO_3_, which were added in equal volume to the supernatant followed by incubation again at 28 ± 2 °C on an orbital-shaker at 150 rpm for 24 h. Color of the reaction mixture was changed from yellow to reddish-brown at 5 mM AgNO_3_ concentration, which was an indication of the formation of AgNPs. Then, the nanoparticle solution was centrifuged again to obtain a pure form of NP-containing cell-free supernatant. After centrifugation, the supernatant was placed at 100 °C in an oven to completely remove the water contents. Finally, the crystals of AgNPs settled at the bottom of the glass beaker were ground into a fine powder, washed with deionized water, and placed again in the oven to obtain the purified powder of AgNPs.

### 4.4. Antibacterial Activity of Biogenic AgNPs

The antimicrobial activity of AgNPs was evaluated by using agar well-diffusion assay, as described by Nanda and Saravanan [46]. The plates were subjected to the formation of five wells of 5 mm diameter with the help of sterile well borer. Four concentrations (5, 10, 15, and 20 µg mL^−1^) of AgNP suspension (20 µL each) were poured into the corresponding wells in triplicate. For this purpose, corresponding milligrams of AgNPs were measured on electronic balance suspended in 1 L volume of deionized water. The suspensions were further homogenized by sonication, followed by the addition of 20 µL from each of these suspetions to the agar wells. The control sample (0 µg mL^−1^ AgNPs) carried 20 µL deionized water in the central well of Petri plates. The plates were incubated at 28 ± 2 °C for 48 h. The diameter of the inhibition zone, representing the antibacterial activity, was measured edge-to-edge across the center of the disk. Similarly, the MIC of biogenic AgNPs against *Xoo* was determined in 96-well microtiter plates according to Ibrahim et al. [33]. The overnight *Xoo* culture was adjusted to 10^8^ CFU mL^−1^ in nutrient broth media. A total of 200 µL of NB media was mixed with 10 µL of bacterial culture and four concentrations (5, 10, 15, and 20 µg mL^−1^) of AgNP suspension (20 µL each) were poured into the corresponding wells in triplicate. The wells treated with 20 µL *Xoo* culture were used as the control. The microtiter plates were incubated at 28 ± 2 °C for 48 h. The MIC of AgNPs was determined at OD600 nm by a scanning microplate spectrophotometer (Thermo Fisher Scientific Inc., Waltham, MA, USA).

### 4.5. Characterization of AgNPs

The presence of AgNPs in the culture supernatant was confirmed by using the UV/visible spectroscopy at a wavelength range of 300–600 nm in order to check the maximum absorbance [47]. 

The FTIR (ATR-FTIR, BRUKER, USA) spectral analysis was used for the identification of functional groups and capping proteins in the silver nanoparticles, which are responsible for the constancy and stability of the AgNPs. For FTIR analysis, the powder form of the biogenic AgNPs was mixed with potassium bromide, and spectra were observed by FTIR spectrum analysis [48].

The X-ray diffraction patterns of biogenic AgNPs were confirmed by the previously described method of Shankar et al. [49]. For XRD pattern determination, the drop of AgNPs suspension was coated over a glass substrate and placed in the X-ray diffractometer apparatus (STOE, Germany) working at 45 kV voltage and 40 mA of current with Ag Kα rays. The samples were scanned at a step size of 0.04, step time of 0.5s/step, and 2θ ranged from 10° to 80°. The wavelength of CuKα radiation was 1.540 Å [50]. The structural crystallinity was analyzed manually, and the Debye–Scherrer’s formula (D = Kλ/β cosθ) was used to calculate the average particle size, where D is crystallite size (nm), K is Sharrer’s constant (0.9), λ is wavelength of X-ray source, β is full width at half maximum of reflection peaks (radians), and θ is peak position (radians).

The morphological shape and surface characteristics of AgNPs were investigated by using scanning electron microscopy (TM-1000, Hitachi, Japan). The slides were prepared by adding an aliquot of AgNPs and fixing the coverslip on aluminium stub by using double-sided adhesive carbon tape followed by observation by SEM, as previously described by [51]. The confirmation of the metallic fraction of the samples was executed with energy-dispersive spectroscopy (EDS). 

The particle shape and morphology of AgNPs were characterized by using transmission electron microscopy (JEM-1230, JEOL, Akishima, Japan), according to Das et al. [52]. For TEM analysis, the carbon-coated grid was loaded with a drop of diluted nanoparticles and subjected to analysis. SEM, TEM, and EDS analysis of biogenic AgNPs were carried out at the Center of Electron Microscopy, Life Sciences Division, Zhejiang University, China. 

### 4.6. Experimental Design of the Pot Plants

Biogenic AgNPs were used to evaluate the efficacy to control symptoms caused by BLB disease infestation on rice plants in a pot experiment under controlled environmental conditions. Rice seeds (cv. Super Basmati) were surface-sterilized and grown, followed by filling of 15 seeds per pot with sterilized peat moss and soil in equal amount. The experiments were repeated three times, and each treatment had three replications. The rice seedlings were grown in pots (20 cm) and placed in the plant growth chamber under optimum conditions (temperature of 27 to 30 °C and humidity of 70% to 90%). At the three-leaf stage after 7 days, the leaves were injured with the help of sterile needles to facilitate the pathogen entry. The pathogen was cultured separately at a cell density of 10^8^ cfu mL^−1^, and cells were separated through centrifugation. The cell pellet was washed and suspended in the same volume of saline solution (0.85% NaCl). The pathogen inoculum was sprayed on the plants with the help of a sprinkler sprayer. The control healthy plants were sprayed with sterile distilled water. Both diseased and healthy plants were continuously supplied with an equal volume of half strength Hoagland solution whenever required. After 15 days, the appearance of clear disease symptoms was observed in the form of water-soaked streaks near the leaf tips and the plants were treated with the suspensions of AgNPs. Both diseased and healthy plants received 25, 50, 75, and 100 mg L^−1^ suspensions of AgNPs along with the sterile distilled water in place of AgNP suspension in control treatments. The lesion length and inhibition rate of the inoculated leaves were measured after 15 days of appearance of disease symptoms in the form of percentage inhibition by the formula given below [53]. Relative lesion length (%) = Total lesion length/Total leaf length × 100.

### 4.7. Measurement of Plant Parameters

Growth parameters to evaluate the effect of AgNPs on rice growth and biomass were measured in the form of root and shoot length as well as fresh and dry weight by using standard protocols. The physiological parameters were determined by measuring the content of the malondialdehyde (MDA) and H_2_O_2_, proline content, catalase activity, peroxidase activity, and total phenolic contents. 

The plant tissues were examined to determine the MDA content by using the thiobarbituric acid (TBA) method described by Heath and Packer [54]. The H_2_O_2_ content was measured by using a previously described method by Jana and Choudhuri [55]. For this, the homogenized sample was centrifuged and incubated according to the standard protocols. The absorbance was measured at 410 nm using Ultrospec 3000 (Biochrom Ltd., Cambridge, UK) spectrophotometer.

The proline content in leaf tissues of rice plants was measured according to a method described by Bates et al. [56] by the same spectrophotometer described above.

The catalase activity of supernatant was determined by using a protocol described by Aebi [57], and the peroxidase activity in homogenized tissues was measured by the method described by Chance and Maehly [58]. 

The total phenolic contents were determined on the basis of the method of Julkunen-Tiitto [59] with few modifications. The fresh leaf tissues (0.5 g) were ground (80% *v*/*v* acetone) and spinned at 4 °C. The 100 µL of the supernatant liquid was dissolved in 2 mL water and 1 mL of Folin–Ciocalteau phenol-reagent, followed by the addition of 5 mL of sodium carbonate (20% *w*/*v*) and ddH_2_O was added to make a final volume of up to 10 mL. The absorbance was recorded by using a UV-VIS spectrophotometer at 750 nm.

### 4.8. Statistical Analysis

Statistix (version 8.1) software was run to perform analysis of variance. The least significant difference test (Fisher’s LSD) at 95% confidence level was performed to determine the differences in the treatments’ means.

## 5. Conclusions

In conclusion, this study successfully isolated an AgNO_3_-resistant bacterial strain SZT1 from wastewater-contaminated soil, which was further identified as *B. cereus* by phylogenetic analysis. Using the strain, we carried out an extracellular biosynthesis of AgNPs, which was confirmed on the basis of the maximum absorption peak at 418.99 nm in UV/VIS spectral analysis. The biogenic AgNPs demonstrated strong in vitro and in vivo antibacterial activity against rice bacterial pathogen *Xoo* along with increase in growth and cellular antioxidant enzyme concentrations. The characterized AgNPs were found to be of spherical shapes with particle size ranging from 18 to 39 nm. The nanoparticles were stabilized by protein functional groups in bacterial culture mixture. Moreover, the biogenic AgNPs showed no toxicity to healthy rice plants. Overall, these results suggested that the application of green AgNPs is an effective and environmentally safe strategy to control BLB disease in rice plants.

## Figures and Tables

**Table 1:** The physico-chemical analysis of wastewater-contaminated soil.

Soil Properties	Value	Soil Properties	Value
EC (dS m^−1^)	8.78	Available K (mg kg^−1^)	142
pH	8.5	Ag (mg kg^−1^)	65
Organic matter (%)	1.43	Zn (mg kg^−1^)	211
Organic C (g kg^−1^)	4.32	Cd (mg kg^−1^)	6.9
Total N (g kg^−1^)	0.62	Ni (mg kg^−1^)	104
Available P (mg kg^−1^)	4.3	Pb (mg kg^−1^)	273

**Table 2:** Effect of AgNPs on lesion length and inhibition rate of *Xanthomonas oryzae* pv. *oryzae* (*Xoo*).

Treatments ∗	Lesion Length (cm)	Inhibition Rate %
Diseased Rice Plants		
Control 2	8.64 (0.50)^a^	-
25 mg L^−1^	5.92 (0.70)^b^	31.36 (8.72)^c^
50 mg L^−1^	4.40 (0.40)^c^	49.12 (1.73)^b^
75 mg L^−1^	3.49 (0.33)^d^	59.34 (6.13)^b^
100 mg L^−1^	2.38 (0.34)^e^	72.51 (2.34)^a^

∗ Data are presented as mean of three replicates (*n* = 3) with standard errors in parentheses. Significance among treatment means is presented as the different lower-case letters (Fisher’s LSD; *p* ≤ 0.05).

**Table 3:** Effect of AgNPs on growth parameters of healthy and diseased rice plants in a pot experiment.

Treatments ∗	Root Length (cm)	Shoot Length (cm)	Root FW (mg)	Shoot FW (mg)	Root DW (mg)	Shoot DW (mg)
**Healthy Rice Plants**						
Control 1	5.44 (0.28)^c^	26.99 (0.45)^ab^	140.30 (1.95)^a^	794.59 (28.16)^a^	40.43 (1.90)^bc^	158.88 (10.22)^a^
25 mg L^−1^	5.87 (0.64)^bc^	27.04 (0.46)^ab^	140.77 (3.67)^a^	795.78 (27.56)^a^	42.43 (3.83)^abc^	161.38 (9.26)^a^
50 mg L^−1^	6.03 (0.38)^abc^	28.07 (0.97)^a^	143.07 (6.42)^a^	806.52 (15.73)^a^	43.50 (3.90)^ab^	161.52 (8.90)^a^
75 mg L^−1^	6.14 (0.03)^ab^	28.19 (0.95)^a^	143.16 (4.59)^a^	808.56 (16.39)^a^	44.24 (5.58)^ab^	164.68 (11.12)^a^
100 mg L^−1^	6.32 (0.10)^ab^	28.25 (0.77)^a^	143.21 (4.47)^a^	808.39 (16.55)^a^	44.97 (2.79)^a^	165.14 (9.54)^a^
**Diseased Rice Plants**						
Control 2	3.47 (0.26)^e^	21.64 (1.00)^d^	92.40 (4.32)^d^	539.47 (23.20)^e^	20.72 (1.50)^e^	123.52 (5.81)^c^
25 mg L^−1^	4.22 (0.11)^d^	23.52 (0.88)^c^	99.49 (5.09)^cd^	590.32 (26.72)^d^	24.26 (2.72)^e^	134.94 (5.71)^bc^
50 mg L^−1^	4.80 (0.52)^d^	26.17 (1.31)^b^	107.31 (6.35)^c^	626.02 (25.39)^c^	29.05 (1.95)^d^	137.17 (4.07)^b^
75 mg L^−1^	5.41 (0.30)^c^	26.67 (0.49)^b^	128.21 (10.23)^b^	725.81 (35.21)^b^	32.72 (1.40)^d^	153.51 (5.35)^a^
100 mg L^−1^	6.59 (0.37)^a^	27.17 (0.69)^ab^	138.27 (5.06)^a^	790.21 (39.03)^a^	39.39 (2.01)^c^	156.51 (5.79)^a^

∗ Data are presented as mean of three replicates (*n* = 3) with standard errors in parentheses. Significance among treatment means is presented as the different lower-case letters (Fisher’s LSD; *p* ≤ 0.05).

**Table 4:** Effect of AgNPs on physiological parameters of healthy and diseased rice plants in a pot experiment.

Treatments ∗	MDA (ƞmol g^−1^ DW)	H_2_O_2_ (ƞg g^−1^ DW)	Proline (µg g^−1^ DW)	CAT (U mg^−1^ protein)	POD (U mg^−1^ protein)	Total Phenolics (mg g^−1^ FW)
**Healthy Rice Plants**						
Control 1 (none)	3.85 (0.23)^b^	12.58 (1.09)^d^	2.38 (0.10)^c^	26.99 (0.87)^f^	24.14 (0.62)^e^	17.73 (1.32)^h^
25 mg L^−1^	3.22 (0.18)^cd^	11.56 (0.20)^e^	2.81 (0.19)^bc^	27.72 (1.68)^f^	26.79 (1.50)^d^	24.26 (1.64)^f^
50 mg L^−1^	3.08 (0.07)^d^	9.72 (0.19)^ef^	3.64 (0.23)^a^	33.30 (1.56)^de^	28.12 (1.54)^d^	32.34 (1.07)^d^
75 mg L^−1^	2.15 (0.13)^e^	8.86 (0.12)^f^	3.65 (0.15)^a^	37.96 (1.66)^b^	34.13 (0.59)^ab^	37.66 (1.02)^c^
100 mg L^−1^	1.45 (0.08)^f^	8.39 (0.24)^g^	3.79 (0.15)^a^	43.12 (1.54)^a^	35.15 (0.54)^a^	42.90 (1.11)^a^
**Diseased Rice Plants**						
Control 2 (pathogen)	4.73 (0.40)^a^	21.64 (1.00)^a^	2.42 (0.37)^c^	22.56 (1.32)^g^	21.64 (1.00)^f^	18.73 (1.15)^gh^
25 mg L^−1^	4.58 (0.23)^a^	18.03 (0.65)^b^	2.57 (0.27)^bc^	23.75 (1.15)^g^	23.40 (1.01)^e^	20.52 (1.06)^g^
50 mg L^−1^	3.51 (0.06)^bc^	14.78 (0.53)^c^	2.60 (0.07)^bc^	30.99 (1.48)^e^	28.18 (0.52)^d^	27.31 (1.20)^e^
75 mg L^−1^	3.11 (0.11)^c^	13.24 (0.78)^d^	2.68 (0.26)^bc^	34.36 (1.06)^cd^	32.18 (1.21)^c^	32.42 (0.95)^d^
100 mg L^−1^	2.43 (0.17)^e^	8.63 (0.43)^fg^	2.98 (0.71)^b^	36.09 (1.50)^bc^	33.14 (0.57)^bc^	39.99 (1.48)^b^

∗ Data are presented as mean of three replicates (*n* = 3) with standard errors in parentheses. Significance among treatment means is presented as the different lower-case letters (Fisher’s LSD; *p* ≤ 0.05).
